# Acceleration of Electrons by Whistler‐Mode Hiss Waves at Saturn

**DOI:** 10.1029/2021GL096213

**Published:** 2022-01-27

**Authors:** E. E. Woodfield, S. A. Glauert, J. D. Menietti, R. B. Horne, A. J. Kavanagh, Y. Y. Shprits

**Affiliations:** ^1^ British Antarctic Survey Cambridge UK; ^2^ University of Iowa Iowa IA USA; ^3^ GFZ Potsdam Germany; ^4^ UCLA Los Angeles CA USA

**Keywords:** Saturn, radiation belt, wave‐particle interactions

## Abstract

Plasmaspheric hiss waves at the Earth are well known for causing losses of electrons from the radiation belts through wave particle interactions. At Saturn, however, we show that the different plasma density environment leads to acceleration of the electrons rather than loss. The ratio of plasma frequency to electron gyrofrequency frequently falls below one creating conditions for hiss to accelerate electrons. The location of hiss at high latitudes (>25°) coincides very well with this region of very low density. The interaction between electrons and hiss only occurs at these higher latitudes, therefore the acceleration is limited to mid to low pitch angles leading to butterfly pitch angle distributions. The hiss is typically an order of magnitude stronger than chorus at Saturn and the resulting acceleration is rapid, approaching steady state in one day at 0.4 MeV at L = 7 and the effect is stronger with increasing L‐shell.

## Key Points

- Low ratio of plasma to gyro‐frequency causes whistler‐mode hiss to accelerate rather than scatter electrons
- Hiss acceleration at high latitudes may lead to butterfly electron pitch angle distributions
- Rapid acceleration over a timescale of hours which strengthens with increasing L‐shell

## 1 Introduction

The importance of whistler‐mode chorus in locally accelerating electrons is well known at the Earth (Horne et al., 2005; Reeves et al., 2013) and has recently been found to be important at Jupiter and Saturn (Horne et al., 2008; Woodfield et al., 2014, 2019). Whilst chorus is a plasma wave observed in discrete rising or falling tones observed in two frequency bands separated by half the electron gyrofrequency (Li et al., 2011), whistler‐mode hiss is a broadband emission at the lower end of the whistler‐mode frequency range (10 Hz to several kilohertz; Li et al., 2015) which occurs in the high density plasmasphere (Meredith et al., 2018; Hartley et al., 2018). At the Earth hiss waves are well known for scattering electrons from the radiation belts into the atmosphere and are thought to be a source of diffuse aurora (Thorne, 2010; Li et al., 2015). Since hiss does not accelerate electrons in the terrestrial environment it has never been considered as a candidate for acceleration in planetary magnetospheres. However, the presence of very low density regions at Saturn coincident with observed hiss waves are highly likely to produce significant electron acceleration in a similar manner to chorus waves (Woodfield et al., 2019). This work assesses the effect of hiss on electrons at Saturn for the first time.

Hiss is observed at Saturn (Menietti et al., 2019, 2020) in the form of diffuse emissions and also as the funnel shaped auroral hiss. Auroral hiss can be found near the footprints of field aligned currents, plasma injections, reconnected field lines, and field lines that connect to the moons, for example, Enceladus (Bader et al., 2019; Menietti et al., 2019, 2020; Palmaerts et al., 2016; Sulaiman et al., 2018). Kronian hiss is thought to be generated in a similar manner to auroral hiss in the Terrestrial system by upgoing electron beams (Maggs, 1976) with energy of the order of 100s eV. Hiss intensity at Saturn is at least an order of magnitude larger than that of chorus and typically exhibits a larger wave normal angle.

Previous studies of the cyclotron resonant interaction with chorus at Saturn found that strong electron acceleration occurs when chorus interacts with electrons in a very low density plasma region where the ratio of the plasma to electron gyrofrequency, *f*_*pe*_/*f*_*ce*_, is less than one. Woodfield et al. (2019) used a new chorus intensity survey in the region 2.5*R*_*S*_–5.5*R*_*S*_, combined with the density model of (Persoon et al., 2015), and found evidence for strong acceleration through chorus‐electron Doppler‐shifted resonant wave‐particle interactions inside of ≈5.5*R*_*S*_. This is in stark contrast to regions outside ≈5.5*R*_*S*_ where *f*_*pe*_/*f*_*ce*_ > 1 for a significant range of latitudes where chorus is observed and acceleration is relatively slow.

Due to the scale height of the Enceladus torus, a low‐density region (*f*_*pe*_/*f*_*ce*_ < 1) begins at typically 10°–20° latitude. The latitudinal dependence of the density leads to a strong pitch angle dependence in the diffusion coefficients. The simulations of Woodfield et al. (2019) demonstrated how this pitch angle dependence should manifest as butterfly pitch angle distributions (PADs) that flatten over a period of a few days at 100s keV, tens of days at MeV energies. Butterfly PADs have a minimum at 90° and maxima around 30–40°, field aligned PADs have a minimum at 90° and rapid increases close to 0 and 180° (Clark et al., 2014).

Whistler‐mode waves continue to even higher latitudes in the form of hiss waves rather than chorus (Menietti et al., 2019) where *f*_*pe*_/*f*_*ce*_ is almost exclusively less than one. In this study we test if the same form of strong, pitch angle dependent acceleration is found.

## 2 Data and Method

### 2.1 Wave and Density Data

In order to model the electron‐hiss interaction we need details of the waves and the plasma environment. A detailed survey of hiss at Saturn using 11 years of Cassini data was published by Menietti et al. (2019). Hiss waves were selected by limiting the frequency to greater than the lower hybrid frequency and less than 3.5 kHz (to avoid contamination from higher frequency radio emission and electrostatic waves; hiss and chorus are very rarely observed above this frequency at Saturn). A minimum power threshold of 10^−10^ V^2^m^−2^Hz^−1^ was chosen through extensive inspection of frequency spectrograms to remove noise and also chorus or Z‐mode emission that may occasionally overlap the hiss frequency range but with lower wave power levels than the hiss. There is a natural separation between hiss and chorus observations which occurs at 25° latitude with the vast majority of chorus (hiss) occurring below (above) this latitude. This has been established through extensive visual inspection of the data and characterized in the 25° cutoff threshold used in Menietti et al. (2014, 2019). We consider the hiss survey data on a global scale by averaging the wave power over a full drift of the electrons around the planet and therefore assume that there is no variation with local time.

**Figure 1:**
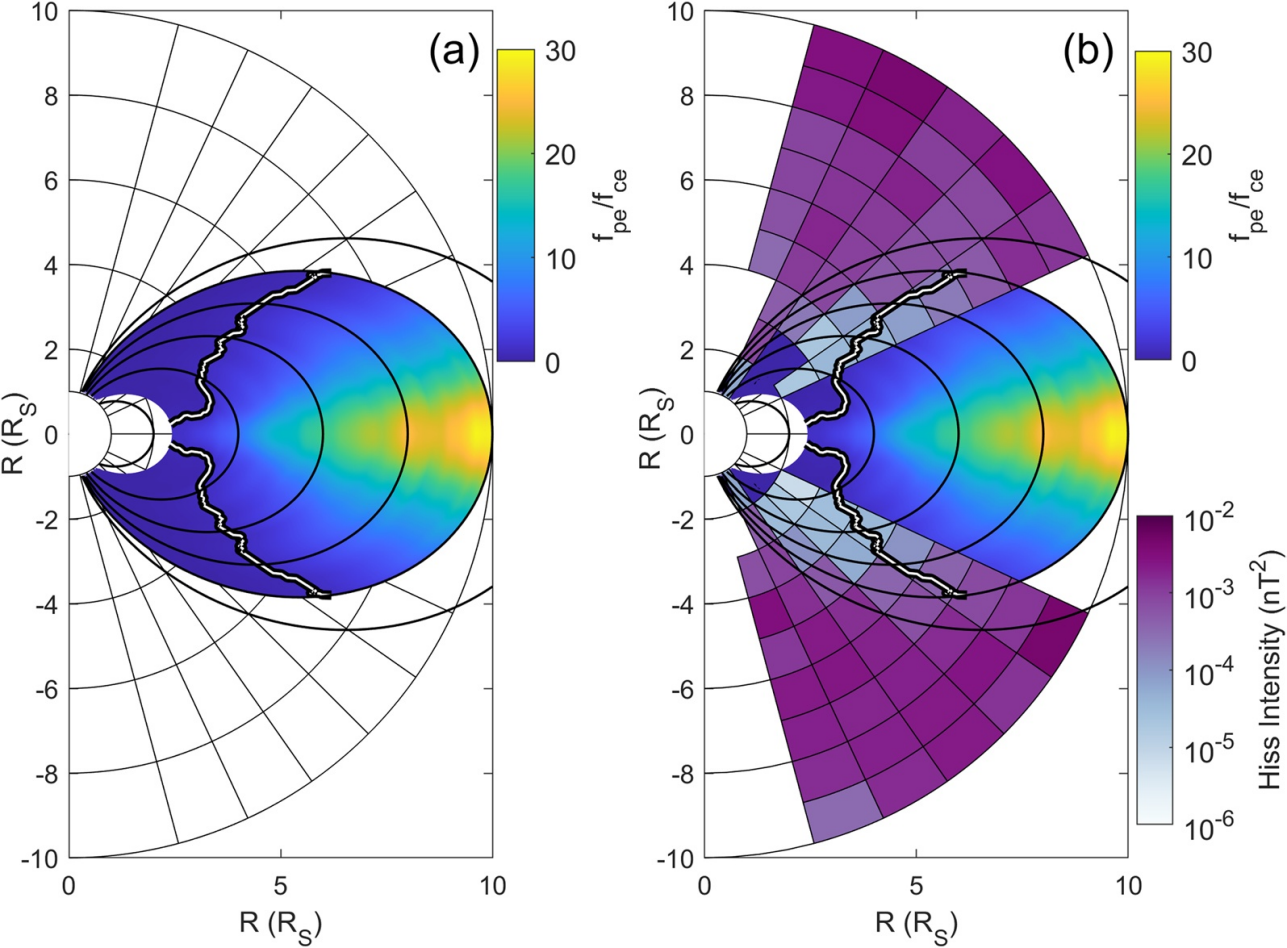
(a) Meridional cut of ratio of plasma to gyrofrequency (blue to yellow color scale). White on black line indicates *f*_*pe*_/*f*_*ce*_ = 1. Black oval lines indicate dipole magnetic field lines. (b) As for (a) but with hiss wave intensity from Menietti et al. (2019) overlaid (purple color scale).

Figure 1 shows the hiss wave intensity from Menietti et al. (2019) (starting at 25° latitude in the purple color scale) overlaid on *f*_*pe*_/*f*_*ce*_ (blue to yellow color scale). The gyro‐frequency is calculated using a centered dipole magnetic field (equatorial surface magnetic field strength 2.1951 × 10^−5^ T) and the plasma frequency is calculated by combining electron density models from the inner magnetosphere (Persoon et al., 2020) and ionosphere (Persoon et al., 2019; the larger of the two values of density is used where they overlap close to the planet). The white on black line shows the latitude boundary above which *f*_*pe*_/*f*_*ce*_ < 1. The majority of the hiss emission is in this region where *f*_*pe*_/*f*_*ce*_ < 1.

The wave intensity, *p* (in nT^2^), does not vary greatly with latitude along each magnetic L‐shell line (black oval lines in Figure 1), we have therefore fitted an equation to the wave intensity that is only dependent on L‐shell and not on latitude using the data from Menietti et al. (2019; figure 6).

(1) $$\left\{ \begin{array}{cc} p=10^{\left(0.2431L-5.72\right)} & 2<L<12\\ p=1.66\times 10^{-3} & L\geq 12 \end{array}\right.$$

The quasi‐linear approach typically uses averaged inputs for the parameters required to calculate the diffusion coefficients and we take that approach here. We fitted a Gaussian of the form in Equation 2 (where *x* is the variable of interest) to the mean frequency data in Menietti et al. (2019) and find *f*_peak_ = 100 Hz, *f*_width_ = 4,000 Hz with a lower and upper cutoff of 100 and 3,500 Hz respectively.

(2) $$p\left(x\right)=p_{0x}e^{-{\left(x-x_{peak}\right)}^{2}/x_{width}^{2}}$$

The wave normal angle, *ψ*, is characterized using *x* = tan(*ψ*) in Equation 2 (Glauert & Horne, 2005) with a value of *ψ*_peak_ = 60° taken from Menietti et al. (2019) and we assume the commonly used value of *ψ*_width_ = 15°, with cutoffs at 30° and 80°. In both cases *p*_0*x*_ is defined using the formula in Equation 1.

### 2.2 Diffusion Coefficients and the 2D Model

Resonant wave‐particle interactions can be assessed using quasi‐linear theory and the effects formed into pitch angle and energy diffusion coefficients (Kennel & Petschek, 1966). To isolate the effect of the wave‐particle interaction the modified Fokker‐Planck equation without radial diffusion is solved (Equation 3) (Glauert et al., 2014; Woodfield et al., 2019).

(3) $$\begin{array}{ccc} \frac{\partial f}{\partial t} & = & \frac{1}{g(\alpha )}{\left.\frac{\partial }{\partial \alpha }\right|}_{E,L}g(\alpha )\left(D_{\alpha \alpha }{\left.\frac{\partial f}{\partial \alpha }\right|}_{E,L}+D_{\alpha E}{\left.\frac{\partial f}{\partial E}\right|}_{\alpha ,L}\right)\\ & & +\frac{1}{A(E)}{\left.\frac{\partial }{\partial E}\right|}_{\alpha ,L}A(E)\left(D_{EE}{\left.\frac{\partial f}{\partial E}\right|}_{\alpha ,L}+D_{E\alpha }{\left.\frac{\partial f}{\partial \alpha }\right|}_{E,L}\right)-\frac{f}{\tau } \end{array}$$

*f* is the particle phase space density, *t* is time, *E* is the relativistic kinetic energy, *E*_0_ is the electron rest mass energy, *α* is the equatorial pitch angle. *g*(*α*) and *A*(*E*) are given by

(4) $$g\left(\alpha \right)=\sin\;2\alpha \left(1.3802-0.3198\left(\sin\;\alpha +\sqrt{\sin\;\alpha }\right)\right)$$

(5) $$A\left(E\right)=\left(E+E_{0}\right)\sqrt{\left(E\left(E+2E_{◦}\right)\right)}$$

*D*_αα_ is the bounce‐averaged pitch angle diffusion coefficient responsible for changing the pitch angle. Pitch angle diffusion is coupled to energy diffusion, *D*_EE_, which is described through the cross diffusion coefficients *D*_*αE*_ = *D*_*Eα*_. *τ* is the atmospheric loss timescale.

Loss to the atmosphere has been included as pitch angle diffusion based on the work of Abel and Thorne (1998). The atmospheric data on density and temperature for Saturn is from (Moore et al., 2009) and more details can be found in the supplementary information of Woodfield et al. (2019). All other collision effects have been excluded (moons, rings, neutral torus, and plasma torus) so as to isolate the effect of the waves.

The diffusion coefficients are calculated using the PADIE code (Glauert & Horne, 2005) using the wave data, magnetic field and plasma density described in the previous section. The interaction range of the waves was limited to 25°–50° with a latitude resolution on the wave power of 2°.

We have run the BAS Radiation Belt Model (BAS‐RBM; Glauert et al., 2014) for Equation 3 without any radial diffusion to show the local effect of the hiss waves. The grid used was 900 × 900 points for *α* and log_*e*_(*E*) with a timestep of 10 s. The minimum and maximum energy were 40 keV and 15 MeV. We want to compare the effect of the hiss waves at different L‐shells without confusing that effect with changes in the initial condition that would be introduced by using a data based initial grid and boundary conditions. We therefore use a theoretical initial grid given by Equation 6 which simulates an empty radiation belt with a pitch angle gently peaked near 90° (Woodfield et al., 2014). The boundary at *α* = 90° is set to *∂f*/*∂α* = 0 and *f* = 0 at *α* = 0°.

(6) $$j=j_{0}\sin^{2}\left(\alpha \right)e^{\left(-\left(E-1.0\right)/0.1\right)}$$

where *E* is in MeV and *j*_0_ = 10^6^ cm^−2^s^−1^sr^−1^MeV^−1^.

## 3 Simulation Results

**Figure 2:**
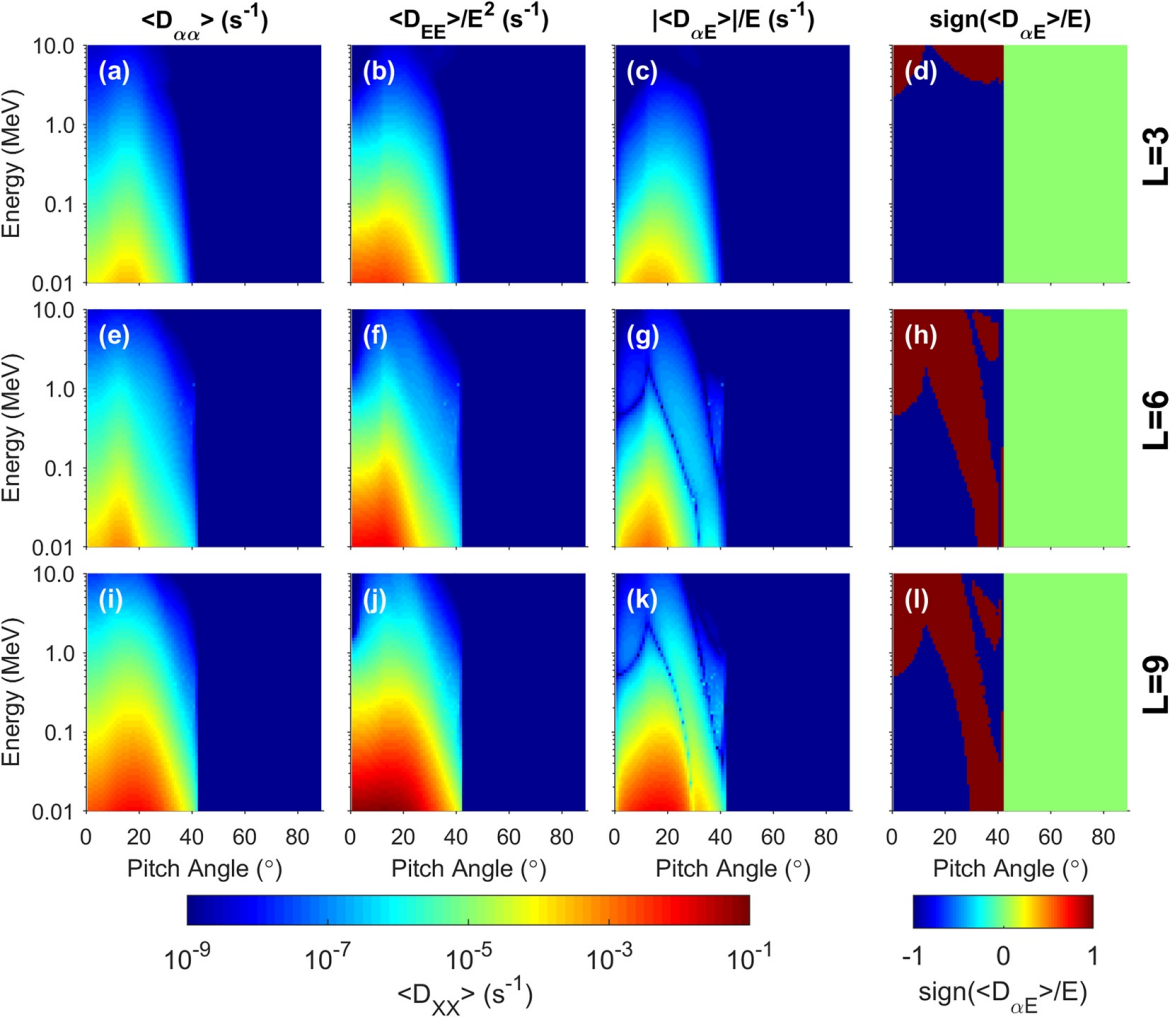
Bounce and Drift averaged diffusion coefficients at L = 3,6,9 (top to bottom rows respectively). (a,e,i) <*D*_αα_>, (b,f,j) <*D*_EE_>/*E* ^2^, (c,g,k) |<*D*_*αE*_>|/*E*, and (d,h,l) sign of <*D*_*αE*_>/*E*.

Figure 2 shows a subset of the calculated diffusion coefficients inside L = 10. Note that no interaction occurs above a pitch angle of ∼42° because the hiss waves are assumed to exist only above 25° latitude in line with observations. Any interaction that occurs will be restricted to closer to the field aligned direction. Note the generally stronger energy diffusion compared to the pitch angle diffusion.

**Figure 3:**
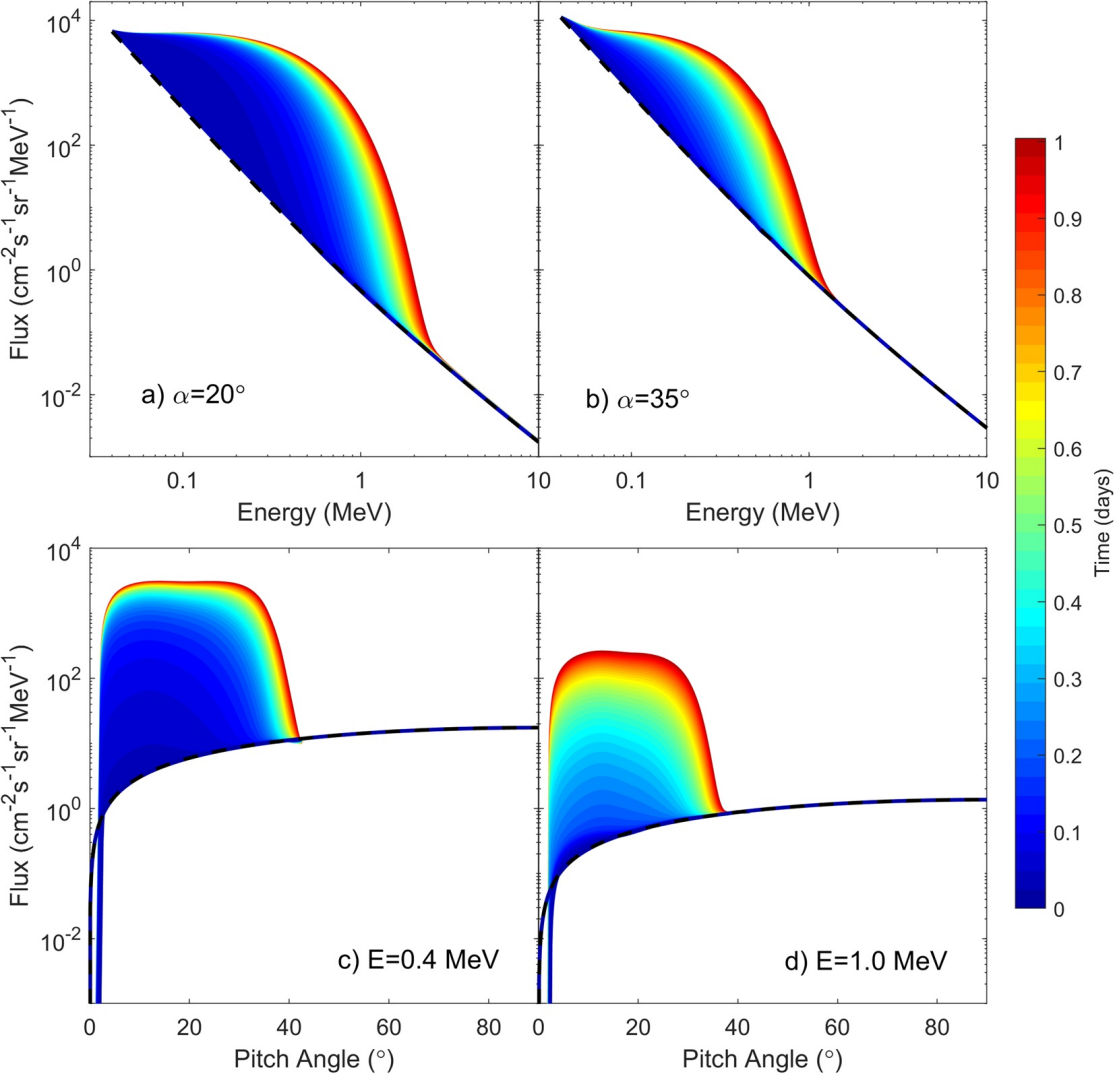
Evolution of electron flux over 24 hr at L = 7. (a and b) at constant pitch angles of 20° and 35° and (c and d) pitch angle distributions at constant energy of 0.4 and 1.0 MeV.

Figure 3 shows the results of running BAS‐RBM at L = 7. The top two panels show energy spectra at 2 different pitch angles and strong acceleration of the electrons is observed. The bottom two panels show that this acceleration is restricted to pitch angles closer to the field aligned direction as expected from the diffusion coefficients in Figure 2. The effect of the hiss waves in isolation is therefore to generate butterfly PADs.

**Figure 4:**
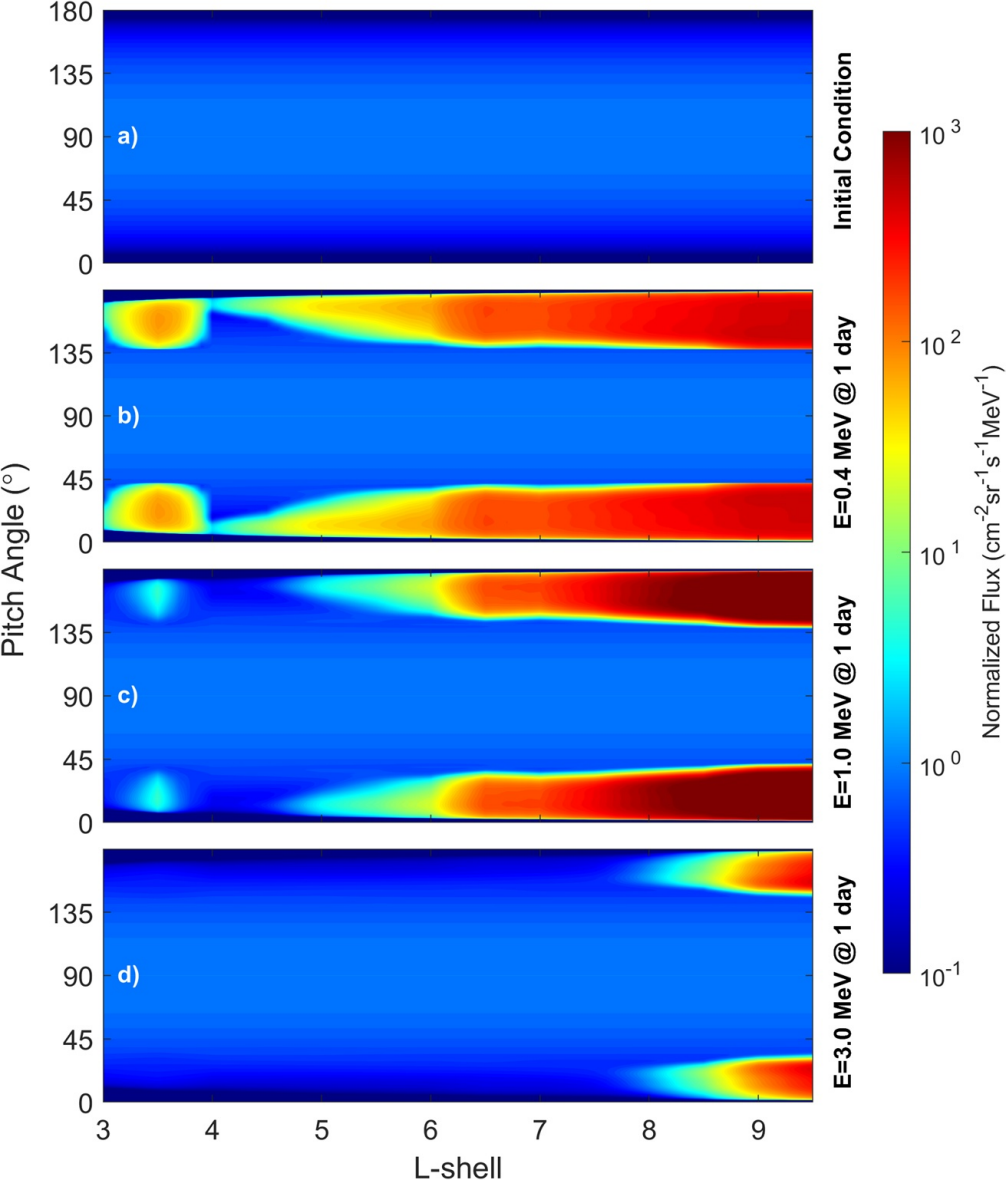
Normalized pitch angle distributions (PADs) from 2D runs run at each L‐shell after 24 hr plotted against L‐shell. (a) Normalized initial conditions for all energies, (b, c, d) normalized PADS at 0.4, 1.0 and 3.0 MeV respectively.

The strength of the interaction with the hiss waves varies with L‐shell as shown in Figure 4. This figure is an amalgamation of the PADs from multiple 2D radiation belt simulations run at different L‐shells, there is no radial transport included here. There is a separate model run for every 0.1 step in L‐shell and the PADs for each L‐shell slice are normalized to the value at 90° for that slice for easy comparison. Panel (a) shows the normalized initial condition with the results after one Earth day (24 hr) shown at three different energies in the remaining panels (b: 0.4 MeV, c: 1.0 MeV, and d: 3.0 MeV).

In general the strength of the electron acceleration increases with increasing L‐shell. This is in line with the general increase in hiss wave intensity reported by Menietti et al. (2019) which continues until the intensity plateaus at around L = 12 and is then maintained past L = 100. There is also a narrow region inside L = 4 where there is increased acceleration at the lowest 2 energies. This is a result of the sudden drop in plasma density inside the orbit of the moon Enceladus bringing *f*_*pe*_/*f*_*ce*_ down and increasing the range of latitudes where the electron hiss interaction is effective.

The rate of electron acceleration is greatest in the 1 MeV case at the largest L‐shells. At 3 MeV, the hiss interacts much less with the electrons but again, most favorably at the highest L‐shells.

## 4 Discussion

Whistler‐mode hiss is a stronger and more diffuse emission than chorus at Saturn and is prevalent where the the plasma density is very low. The presence of a strong interaction between whistler‐mode chorus and electrons in this low density regime has been shown in Woodfield et al. (2019) and the results shown here indicate that the strong acceleration observed is continued as the whistler‐mode emissions change from predominantly distinct chorus to diffuse hiss at around 25° latitude. We split our discussion into three main regions based on the energetic electron observations and dominant physical processes: from the main rings to 4*R*_*S*_, from 4 to 9*R*_*S*_ and outside 9*R*_*S*_.

### 4.1 Between the Main Rings at 2.27*R*_*S*_ and 4*R*_*S*_

At L‐shells close to the planet it has recently been demonstrated that butterfly PADs are observed inside of L = 3.5 at MeV energies (Yuan et al., 2021). This is in line with predictions from previous analysis of Z‐mode and chorus interactions with electrons (Woodfield et al., 2018, 2019). The hiss‐electron interactions reported here also push electron distributions toward butterfly PADs. The strength of the hiss and chorus energy diffusion coefficients at energies over 1 MeV are both slightly larger than those for the Z‐mode and we would expect the effects of these three waves to reinforce each other at high energies.

At energies from 110 to 365 keV the PADs in this region are dominated by pancake shaped distributions peaked at a pitch angle of 90° (Carbary et al., 2011). Somewhere between 365 and 800 keV this changes to butterfly PADs dominating. To reconcile the lower energy pancake PADs with the strong tendency for hiss, chorus and Z‐mode to generate butterfly PADs we note that at these lower energies other influences become more important. For example, the presence of rings and neutral particles from the Enceladus torus will have an increasing influence as energies decrease (Lorenzato et al., 2012). In contrast, Cosmic Ray Albedo Neutron Decay (CRAND) is an important slow and steady source of electrons of 100’s keV energy close to the main rings.

Both radial diffusion (Schulz & Lanzerotti, 1974) and radial adiabatic transport (Hao et al., 2020; Roussos et al., 2018) are expected to be slow in this region. The radial diffusion coefficient is proportional to *L* ^*n*^ where *n* is at least 3 and is thought to be dependent on energy (Clark et al., 2014; Kollmann et al., 2011; Roussos et al., 2007) with a value in the range 10^−12^–10^−6^s^−1^ at L = 3. The effect of radial adiabatic transport decreases away from the corotation drift resonance energy, *E*_CDR_ (where the corotation velocity of the electrons matches and opposes the magnetic gradient and curvature drifts leading to electrons with no net drift). At L = 3, *E*_CDR_ ≈ 2.5 MeV and the the effect of rapid transport is thought to extend several times higher and lower than this value (Hao et al., 2020). Both these processes will increase the equatorial pitch angle of the electrons as they are transported inwards but only by a few degrees at constant first and second adiabatic invariants between 4 and 2.5*R*_*S*_ (Schulz & Lanzerotti, 1974). Yuan et al. (2021) show that the electron MeV PADs just inside of the orbit of Enceladus (L = 3.9) are isotropic or pancake shaped but then rapidly become butterfly PADs inside of approximately 3.5*R*_*S*_. This is the opposite behavior we would expect from both radial diffusion and rapid adiabatic transport further indicating the importance of the wave‐particle interactions inside of 3.5*R*_*S*_.

### 4.2 Between 4 and 9*R*_*S*_

Previous work showed low pitch angle acceleration of electrons due to chorus in low density regions occurs inside L = 5.5 (Woodfield et al., 2019). This mid to low pitch angle acceleration is now extended by the interaction of hiss waves to cover the whole of this region from ∼4 to ∼9*R*_*S*_. In addition, the combination of high density chorus interactions with high latitude Z‐mode waves at L = 6 reported by Yu et al. (2019) results in losses across all pitch angles below a few hundred keV but acceleration above this peaked near 30° due to the Z‐mode waves.

In this region electrons from 20 keV to many MeV are mainly observed with isotropic PADs (Carbary et al., 2011; Clark et al., 2014; Yuan et al., 2021) with butterfly PADs at a few keV (Rymer et al., 2008) and 20–800 keV PADs becoming increasingly butterfly dominated from 7*R*_*S*_ outwards (>800 keV PADs beyond 7*R*_*S*_ were not investigated in; Yuan et al., 2021). Clark et al. (2014) noted that inside 7*R*_*S*_ losses are likely driven by collisions (rings, neutral torus, and plasma torus). Collisions with the neutral torus, plasma torus and ring particles/dust cause slow and continuous losses over a wide range of L‐shells that are important at low electron energies, particularly less than 100 keV (Lorenzato et al., 2012; Woodfield et al., 2018).

Significant losses at MeV energies are expected due to the presence of ion cyclotron waves (Leisner et al., 2011; Meeks & Simon, 2017; Meeks et al., 2016) based on what we know from the Earth and the abundance of these waves due to the pick‐up ion process at Saturn. A common observation from work at the Earth is that the electrons near 90° remain unaffected by ion cyclotron waves (Ross et al., 2020) and this would counteract the acceleration due to hiss, chorus and Z‐mode at low pitch angles potentially resulting in a more isotropic or pancake PAD. This effect will only cover part of this region (limited to inside L = 8 where the ion cyclotron waves are observed).

Radial diffusion and rapid adiabatic transport are both more effective in this region, radial diffusion because L has increased and rapid transport because *E*_*CDR*_ ranges from approximately 1 MeV at L = 4–100s keV at L = 9 and is likely to be a key effect swiftly moving electrons large distances. Including the rapid transport in the traditional diffusion paradigm is non‐trivial and will be the subject of future work. Both of these mechanisms will increase the electron pitch angles as they are moved inwards.

The theoretical initial conditions we use in this study are very helpful in assessing the steady state of the system under specific drivers but in reality the starting electron and pitch angle spectrum, and most importantly the gradients in phase space density on which the waves act will be variable. This will impact the evolution of the spectra over time (Allison et al., 2019). Kollmann et al. (2018) show that electron energy spectra outside of 4*R*_*S*_ are time variable on top of a trend that follows the effect of radial diffusion; some of this variation may be due to hiss and chorus wave acceleration and subsequent losses. It is important to note though, that whatever the starting conditions, the hiss, chorus and Z‐mode will only affect mid to low pitch angles due to the constraints on wave location and the latitude of the low density region.

We have explicitly ignored any processes due to moons in this work because the focus is on the broader trends in electron flux due to only the hiss waves. Standard losses due to absorption of particles by the body of the moon have a very localized effect and indeed the macroscopic moon signatures for electrons are relatively week at Saturn (Roussos et al., 2007). We also note that substantially increased wave activity has been observed at Rhea at L = 8.7 (Santolík et al., 2011) which might also have a significant local effect on the electrons, the investigation of which is beyond the scope of this work.

### 4.3 Outside 9*R*_*S*_

Strong hiss emissions continue out to L > 100 at high latitudes (Menietti et al., 2019) and we suggest that hiss‐electron interactions contribute to the butterfly PADs in the transition region identified by Clark et al. (2014) from 8 to 12*R*_*S*_ and the field aligned PADs in the electrons observed beyond approximately 9*R*_*S*_ (Clark et al., 2014). It is also possible that hiss may play a role in the MeV quasi‐periodic electron injections commonly referred to as QP60 injections (Roussos et al., 2016). The low pitch angle dominated PADs and mapping of these QP60 injections to the main auroral region at Saturn are both suggestive of auroral hiss involvement. It would also be of interest in future work to assess the effect of high latitude wave‐particle acceleration processes, such as that with hiss, on the assumptions behind the Kennel‐Petschek limit (Kennel & Petschek, 1966). For example, it has been noted that the Kennel‐Petschek limit is substantially exceeded at L = 8–10 at Saturn (Tang & Summers, 2012).

## 5 Summary

Hiss at Saturn has a very different interaction with electrons compared to the Earth due to the prevalence of low density regions in the magnetosphere. At Saturn, hiss accelerates electrons and the high latitude nature of where hiss is observed pushes the electron energies upwards at mid to low pitch angles only. These results strengthen the case for wave‐particle acceleration inside 4*R*_*S*_, introduces new possibilities outside 9*R*_*S*_ and increases the need to understand the complex interplay of rapid adiabatic transport, radial diffusion, wave‐particle interactions and collisional losses inbetween.

## Data Availability

Open Research Cassini RPWS data are archived in calibrated, full resolution at the NASA Planetary Data System website (https://pds-atmospheres.nmsu.edu/data_and_services/atmospheres_data/Cassini/inst-rpws.html#finding). Data from Figures 2, 3, 4 are available at the Polar Data Center (PDC) within the British Antartic Survey (https://www.bas.ac.uk/data/uk-pdc/) via doi: https://doi.org/10.5285/c6202511-d70b-45ae-9b72-ff30f4888f5f.
